# Atrial volumetrics and recurrence after catheter ablation for atrial fibrillation

**DOI:** 10.3389/fcvm.2026.1843231

**Published:** 2026-06-22

**Authors:** Hyunho Ryu, Soonil Kwon, Seokmoon Han, Jae-Hyun Kim, Hyo-Jeong Ahn, So-Ryoung Lee, Seil Oh, Eue-Keun Choi

**Affiliations:** 1 Department of Internal Medicine, Seoul National University Hospital, Seoul, Republic of Korea; 2 Department of Internal Medicine, Seoul National University College of Medicine, Seoul, Republic of Korea; 3 Department of Internal Medicine, CHA Bundang Medical Center, CHA University School of Medicine, Seongnam, Republic of Korea

**Keywords:** left atrial appendage, left atrium, recurrent atrial fibrillation, right atrial appendage, right atrium

## Abstract

**Background:**

While left atrial (LA) volume enlargement is a well-known predictor of recurrence after catheter ablation for atrial fibrillation (AF), the association between the recurrence and other atrial volumetrics remains uncertain. This study aimed to assess the association between various atrial volumetrics and the 12-month recurrence of AF after catheter ablation (AFCA).

**Methods:**

Patients with a history of AFCA who underwent pre-procedural cardiac computed tomography were retrospectively included. Atrial volumes were measured using artificial intelligence-based segmentation software (AutoSeg-H). Atrial volumetrics included total LA, LA body, LA appendage (LAA), total right atrium (RA), RA body, and RA appendage (RAA) volumes. AF recurrence was defined as any documented atrial arrhythmia (AA) within 12 months following AFCA. All volumetrics were dichotomized using cutoffs that optimally predicted 12-month AA recurrence on the receiver operating characteristic curve. Cox regression models were used to estimate adjusted hazard ratios (aHRs) of the dichotomized volumetrics.

**Results:**

Among 199 included patients, 45 (22.6%) underwent AA recurrence within 12 months. All atrial volumetrics were significantly higher in the recurrence group than in the non-recurrence group. Optimal cutoffs used for dichotomizing volumetrics were: total LA ≥129.1, LA body ≥112.0, LAA ≥15.3, total RA ≥123.0, RA body ≥82.1, and RAA ≥13.2 (unit: mL). All dichotomized volumetrics, except RAA ≥ 13.2, were significantly associated with recurrence. LAA volume ≥15.3 [aHR 1.97; 95% confidence interval (CI), 1.04–3.74; *P* = 0.038] and RA body volume ≥82.1 (aHR 2.39; 95% CI, 1.03–5.55; *P* = 0.042) were independent predictors for 12-month AA recurrence after AFCA.

**Conclusions:**

Most dichotomized atrial volumetrics were associated with 12-month AA recurrence after AFCA. In particular, LAA ≥15.3 mL and RA body ≥82.1 mL were independently associated with recurrence, suggesting that structural remodeling in these regions may contribute to post-ablation AA recurrence.

## 1 Introduction

Atrial fibrillation (AF) is an arrhythmia characterized by rapid, irregular atrial ectopic activity, and it is the most common cardiac tachyarrhythmia in the general population, with an estimated global prevalence of 59.7 million cases in 2019 (1, 2). AF significantly increases the risk of all-cause mortality, ischemic stroke, and heart failure (3). Because muscle sleeves between pulmonary veins (PVs) and the left atrium (LA) are the predominant source of abnormal ectopic activity that initiates AF (4), pulmonary vein isolation (PVI) with catheter ablation for AF (AFCA) has become a standard therapy for patients who have drug-refractory AF (5).

Despite advances in AFCA, relatively high recurrence rates remain an unresolved issue. Previous studies have reported a 12-month recurrence rate of approximately 35% (6, 7). Among the various risk factors associated with atrial arrhythmia (AA) recurrence after AFCA, numerous studies have explored the impact of atrial volumes on the recurrence. Increased LA volume is associated with a higher risk of recurrence after AFCA (8, 9). Previous studies have also demonstrated a similar association with RA volume (10, 11). Moreover, recent evidence suggests that left atrial appendage (LAA) volume may be independently related to AA recurrence (12, 13).

However, apart from LA volume, the associations between AF recurrence and other atrial volumetrics, such as right atrium (RA), left atrial appendage (LAA), and right atrial appendage (RAA) volume, remain insufficiently established. Furthermore, few studies have directly compared the predictive value of various atrial volumetrics, including total LA, LA body, LAA, total RA, RA body, and RAA volume, for predicting recurrence after AFCA. Most prior investigations have evaluated these parameters individually, without assessing which specific regions provide the strongest prognostic utility. As a result, it remains unclear whether specific volumetric measures outperform others in predicting recurrence after AFCA. Therefore, we aimed to evaluate and compare the predictive value of multiple atrial volumetrics for 12-month AA recurrence following AFCA.

## 2 Methods

### 2.1 Study population

This retrospective, single-center observational study included 220 patients who underwent atrial fibrillation catheter ablation (AFCA) at a tertiary referral center, Seoul National University Hospital (SNUH), Seoul, Republic of Korea, between December 2016 and November 2019. Patients were excluded if they met any of the following criteria: age <19 years; absence of cardiac computed tomography (CT) performed within one week before AFCA; or inadequate cardiac CT image quality for heart chamber segmentation. Of the 220 patients initially identified, eight were excluded due to the lack of pre-procedural cardiac CT imaging, and 13 were excluded because of insufficient image quality for segmentation analysis. Consequently, a total of 199 patients were included in the final analysis. The flowchart for the study is provided in Supplementary Figure S1.

This study adhered to the ethical principles outlined in the Declaration of Helsinki, revised in 2013, and was approved by the Institutional Review Board (IRB) of SNUH (IRB No: H-2201-112-1293). Informed consent was waived due to the retrospective nature of the study.

### 2.2 Cardiac CT image acquisition

Electrocardiography-gated sequential CT imaging was performed using a 256-channel multi-detector CT scanner (SOMATOM Force, Siemens Healthineers, Erlangen, Germany), within one week before AFCA. Image acquisition was synchronized with the systolic phase. A contrast-enhanced scan was obtained following the intravenous administration of 80 mL of iomeprol 400 mg/mL (Iomeron, Bracco, Milan, Italy) at a flow rate of 4 mL/s. End-systolic phase images were reconstructed with a slice thickness of 0.75 mm and an increment interval of 0.5 mm (14).

### 2.3 Protocols for AFCA

The AFCA procedure was performed under deep conscious sedation. After trans-septal punctures, a three-dimensional electroanatomical map of the LA and PV was created using a Pentaray catheter and the CARTO 3 system (Biosense Webster Inc., CA, USA). PVI was achieved through circumferential PV antral ablation using a radiofrequency catheter (Thermocool SmartTouch SF catheter; Biosense Webster Inc., CA, USA). Radiofrequency energy was delivered in power-controlled mode, with 30–40 W (irrigation flow up to 30 mL/min) applied to the anterior and roof segments and 25–40 W (irrigation flow up to 17 mL/min) to the posterior, inferior, and carina segments of PVs. Then, additional ablations, including linear or non-PV trigger ablations or other substrate modifications, were performed at the operator's discretion. In cases where AF persisted despite PVI and adjunctive ablation, electrical cardioversion was subsequently conducted (14–16). More detailed procedural protocols for AFCA are provided in the Supplementary Materials.

### 2.4 Heart chamber segmentation

For each patient, contrast-enhanced cardiac CT images acquired within one week before AFCA, were extracted in DICOM format. Heart chamber segmentation was performed using a dedicated version of the software platform (AutoSeg-H ver. 1.05; AI Medic Inc., Seoul, Republic of Korea). AutoSeg-H is an artificial intelligence-based software designed to reconstruct three-dimensional cardiac anatomy from two-dimensional contrast-enhanced cardiac CT images and segment the heart into individual chambers. AutoSeg-H automatically segmented cardiac chambers from CT images using a deep learning–based semantic segmentation model (17). The model was trained on 516 CT datasets, split into training, validation, and test sets at a ratio of 8:1:1. The CT datasets analyzed in the present study were not included in the model training process.

**Figure 1:**
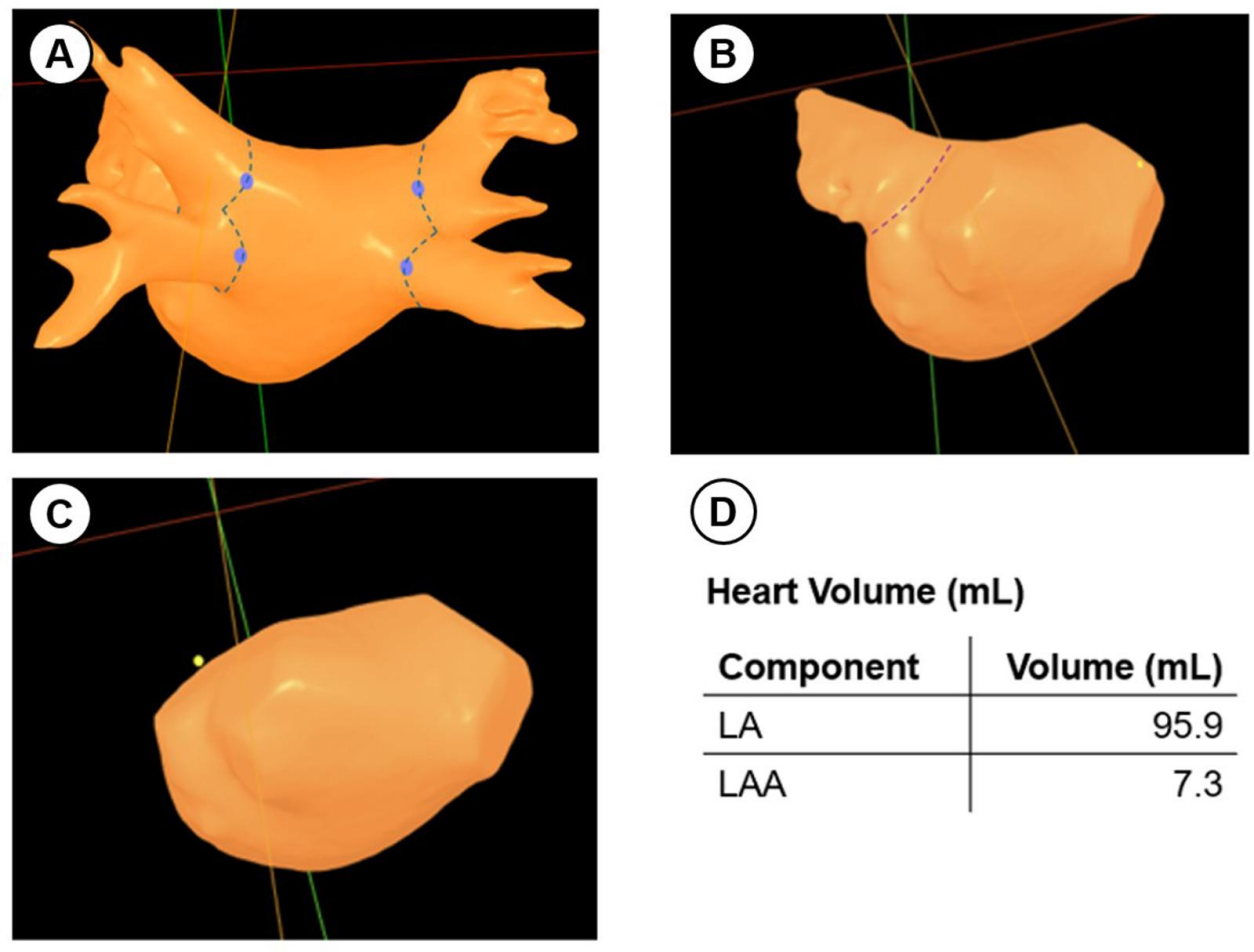
An example of LA segmentation with the AutoSeg-H software. **(A)** Initially reconstructed LA. Blue dotted lines indicate the ostia of PVs. **(B)** LA after PV removal. Purple dotted line indicates the boundary between the LA body and the LAA. **(C)** LA body after LAA removal. **(D)** Automatically calculated LA body and LAA volume using AutoSeg-H. LA, left atrium; LV, left ventricle; RA, right atrium; RV, right ventricle; PV, pulmonary vein; LAA, left atrial appendage.

The software segmented the heart into four chambers: LA, left ventricle (LV), RA, and right ventricle (RV). However, since the LA and RA generated by AutoSeg-H included portions of the PVs and the vena cava, respectively, we conducted additional manual delineation. The boundary between the LA and the PVs was defined at the inflection points formed at their anatomical confluence (18, 19). Similarly, the boundary between the RA and the superior and inferior vena cava (SVC and IVC) was defined at the corresponding anatomical inflection points (19). The remaining portions of LA and RA after excluding the PVs and the SVC/IVC were defined as the total LA and total RA, respectively. Then, LAA and RAA were also manually delineated from the total LA and total RA, with the LA body and RA body defined as the respective residual volumes after subtracting the appendages. The boundary between the atrial body and the appendage was defined at the point of maximal curvature at the junction between the atrial body and the proximal appendage (19, 20) (Figure 1). For manual delineation, H.R. was trained by certified cardiologist S.K., and the delineation process was subsequently performed by H.R. under the supervision of S.K.

### 2.5 Definitions of variables and data collection

Demographic information, past medical history, types of AF (paroxysmal AF or persistent AF), and transthoracic echocardiography parameters at the time of AFCA were collected from the electronic medical record. Past medical history included the CHA₂DS₂-VASc score and comorbidities such as hypertension, diabetes mellitus (DM), heart failure (HF), stroke/transient ischemic attack (TIA), coronary artery disease (CAD), dyslipidemia, and chronic kidney disease (CKD). If a patient had multiple echocardiographic records, the most recent one obtained before AFCA was used.

AA recurrence was defined as a documented episode of AF, atrial flutter, or atrial tachycardia within 365 days after AFCA, confirmed by 12-lead electrocardiography (ECG), 24-hour Holter monitoring, or wearable devices, recorded during emergency department visits, hospitalizations, or outpatient clinic follow-ups. However, episodes occurring within 90 days post-AFCA (blanking period) were not classified as recurrence.

### 2.6 Statistical analysis

Patients were categorized into two groups based on AA recurrence within 12 months after AFCA: the AA recurrence group and the non-recurrence group. Continuous variables were expressed as mean ± standard deviation or median with interquartile range (IQR), depending on the results of normality testing for each variable. Categorical variables were presented as N (%). Comparisons between the AA recurrence and non-recurrence groups were performed using the t-test for continuous variables and the chi-square test or Fisher's exact test for categorical variables.

The optimal cutoff value for each atrial volumetric, maximizing the sum of specificity and sensitivity on the respective receiver operating characteristic (ROC) curve for predicting AA recurrence within one year after AFCA, was determined. Based on these cutoff values, patients were dichotomized into those with atrial volumes greater than or equal to the optimal cutoff and those with atrial volumes below the cutoff. Kaplan–Meier analysis was performed to estimate the 12-month AA recurrence-free survival rate, and differences between the groups were assessed using the log-rank test.

Cox proportional hazards regression analysis was conducted for each dichotomized atrial volumetric to estimate the hazard ratio (HR) with a 95% confidence interval (CI) for 12-month AA recurrence after AFCA. Three models were applied for Cox regression: Model 1 was unadjusted; Model 2 was adjusted for age and sex; and Model 3 was adjusted for age, sex, AF type (paroxysmal AF vs. persistent AF), hypertension, DM, dyslipidemia, HF, and CHA₂DS₂-VASc score. In addition, a multivariable Cox regression model was constructed that included one LA volumetric (total LA, LA body, or LAA) and one RA volumetric (total RA, RA body, or RAA) with the highest HRs, along with the covariates in Model 3, to minimize the effect of collinearity. Variance inflation factor (VIF) values were estimated, and a maximum VIF value of ≥5 in the Cox regression model was considered indicative of collinearity. The proportional hazards assumption was evaluated using Schoenfeld residuals. In addition, to address the potential risk of overfitting due to the limited number of outcome events, a sensitivity analysis using penalized Cox regression with the least absolute shrinkage and selection operator (LASSO) was additionally performed. The optimal penalty parameter was selected using 10-fold cross-validation.

All statistical analyses were performed using R (version 4.4.1; R Core Team, Vienna, Austria). A two-tailed *P*-value of less than 0.05 was considered statistically significant for rejecting the null hypothesis.

## 3 Results

### 3.1 Patient characteristics and atrial volumetrics

Consequently, 199 patients were included in the final analysis, of whom 45 experienced AA recurrence within 12 months after AFCA and 154 did not.

The baseline characteristics of the study population are summarized in Table 1. In the non-recurrence group, the mean age was 60.0 ± 8.5 years, and 116 patients (75.3%) were male. In the AA recurrence group, the mean age was 60.8 ± 8.9 years, and 40 patients (88.9%) were male. The CHA₂DS₂-VASc score did not significantly differ between the two groups [median 2 (IQR 1–3) for both groups, *P* = 0.490]. However, the proportion of patients with persistent AF was significantly higher in the AA recurrence group compared to the non-recurrence group (51.1% vs. 28.6%, *P* = 0.008). Additionally, the prevalence of HF was significantly higher in the recurrence group than in the non-recurrence group (13.3% vs. 3.9%, *P* = 0.030). No significant differences were observed between the groups for other comorbidities. Both groups showed comparable values for LV ejection fraction, LV diastolic internal diameter, and LA anterior-posterior diameter in echocardiography.

**Table 1:** Comparison of the baseline characteristics between patients with and without AA recurrence after AFCA.

Characteristics	Patients without recurrence (*n* = 154)	Patients with recurrence (*n* = 45)	*P*-value
Male	116 (75.3)	40 (88.9)	0.082
Age (year)	60.0 ± 8.5	60.8 ± 8.9	0.399
Current Smoker	12 (7.8)	7 (15.6)	0.119
Current Drinker	56 (36.4)	18 (40.0)	0.657
AF type			0.008
Paroxysmal AF	110 (71.4)	22 (48.9)	
Persistent AF	44 (28.6)	23 (51.1)	
AF duration (month)	23.4 (14.0–49.9)	23.1 (9.1–60.6)	0.486
Hypertension	80 (52.0)	18 (40.0)	0.215
Diabetes mellitus	30 (19.5)	11 (24.4)	0.607
Heart failure	6 (3.9)	6 (13.3)	0.030
Stroke/TIA	13 (8.4)	6 (13.3)	0.386
Coronary artery disease	13 (8.4)	1 (2.2)	0.198
Dyslipidemia	54 (35.1)	13 (28.9)	0.554
Chronic kidney disease	4 (2.6)	3 (6.7)	0.193
CHA_2_DS_2_-VASc score	2 (1–3)	2 (1–3)	0.490
Echocardiography			
LVEF (%)	59.0 (56.0–62.5)	59.0 (56.0–62.3)	0.574
LVIDd (mm)	47.8 ± 3.9	48.6 ± 4.1	0.237
LA AP diameter (mm)	42.3 ± 5.2	43.4 ± 5.0	0.205
AFCA Procedure			0.548
PVI only	82 (53.2)	21 (46.7)	
Additional ablation	72 (46.8)	24 (53.3)	

AA, atrial arrhythmia; AF, atrial fibrillation; TIA, transient ischemic attack; LVEF, left ventricular ejection fraction; LVIDd, left ventricular diastolic internal diameter; LA AP, left atrial anterior-posterior; AFCA, atrial fibrillation catheter ablation; PVI, pulmonary vein isolation.

Continuous variables were expressed as mean ± standard deviation or median with interquartile range (IQR). Categorical variables were presented as N (%).

There was no significant difference in the AFCA procedures performed between patients without AA recurrence and those with recurrence, with additional ablation conducted in 72 patients (46.8%) and 24 patients (53.3%), respectively.

The comparison of atrial volumetrics between the non-recurrence and AA recurrence groups is presented in Table 2. All atrial volumetrics, including total atrial volume, atrial body volume, and appendage volume, were significantly larger in the AA recurrence group than in the non-recurrence group. Specifically, the median total LA volume was 106.5 mL (IQR 82.0–130.5 mL) in the non-recurrence group and 122.7 mL (IQR 96.7–145.5 mL) in the AA recurrence group (*P* = 0.018). The difference in total RA volume between the two groups was even more pronounced, with median total RA volumes of 96.2 mL (IQR 74.5–125.7 mL) and 118.5 mL (IQR 91.2–143.8 mL) in the non-recurrence and AA recurrence groups, respectively (*P* = 0.005).

**Table 2:** Comparison of the atrial volumetrics between patients with and without AA recurrence.

Atrial volumetrics	Patients without recurrence (*n* = 154)	Patients with recurrence (*n* = 45)	*P*-value
Total LA volume (mL)	106.5 (82.0–130.5)	122.7 (96.7–145.5)	0.018
LA body volume (mL)	96.1 (73.0–117.6)	112.0 (89.1–124.7)	0.027
LAA volume (mL)	10.3 (7.2–14.0)	12.1 (7.8–17.6)	0.045
Total RA volume (mL)	96.2 (74.5–125.7)	118.5 (91.2–143.8)	0.005
RA body volume (mL)	87.5 (67.8–115.0)	103.2 (83.5–126.3)	0.012
RAA volume (mL)	9.4 (6.3–12.0)	10.7 (7.1–14.7)	0.027

AA, atrial arrhythmia; LA, left atrium/atrial; LAA, left atrial appendage; RA, right atrium/atrial; RAA, right atrial appendage.

### 3.2 Optimal values of various atrial volumetrics for the prediction of recurrence

ROC curves were generated for each atrial volumetric parameter to evaluate their ability to predict AA recurrence within 12 months after AFCA. Among the volumetrics, total RA volume demonstrated the highest area under the ROC curve (AUROC) at 0.638 (95% CI, 0.550–0.726), whereas LAA volume showed the lowest AUROC at 0.598 (95% CI, 0.496–0.701). The AUROC values and corresponding optimal cutoff values, defined as those maximizing the sum of sensitivity and specificity, are summarized in Supplementary Table S1.

Based on the cutoff value of each volumetric, patients were divided into two groups. For example, patients were categorized as “total LA volume ≥129.1 mL” or “total LA volume <129.1 mL”, and similarly as “total RA volume ≥123.0 mL” or “total RA volume <123.0 mL”.

**Figure 2:**
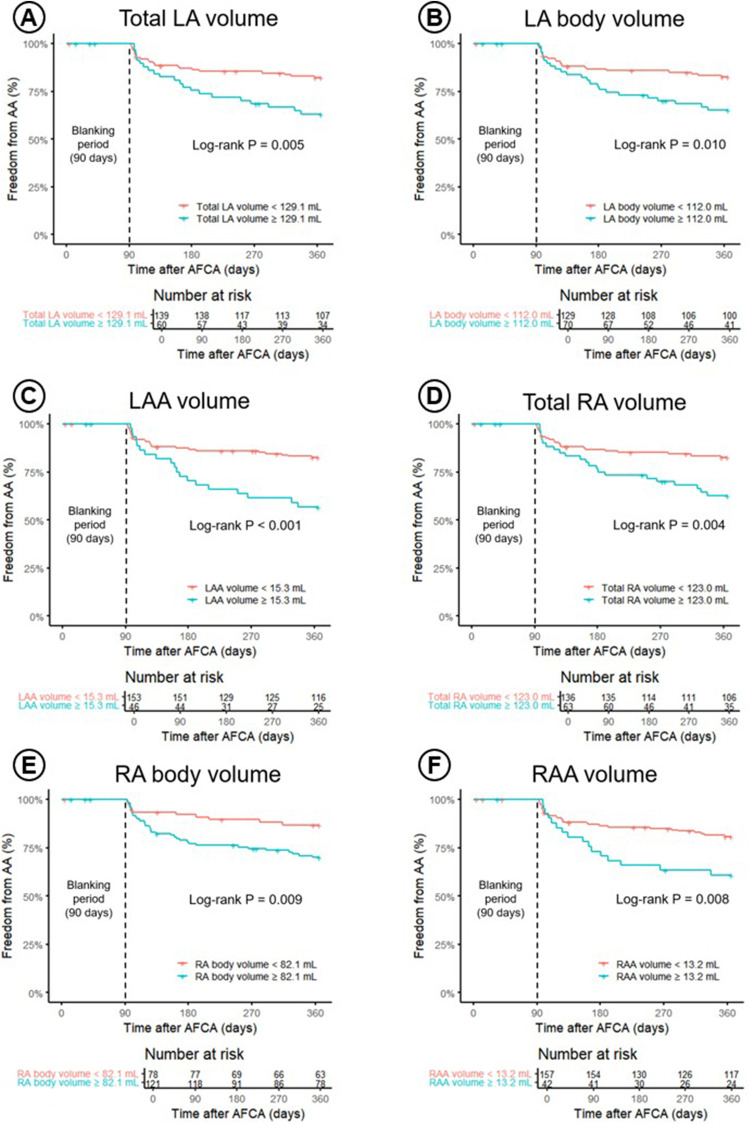
Survival analysis for AA after AFCA. Patients were divided into two groups according to each atrial volumetric based on the optimal cutoff value (defined as the Youden index) for predicting AA recurrence within 1 year after AFCA. Patients grouping according to **(A)** LA volumes, **(B)** LA body volumes, **(C)** LAA volumes, **(D)** total RA volumes, **(E)** RA body volumes, and **(F)** RAA volumes. AA, atrial arrhythmia; AFCA, catheter ablation for atrial fibrillation; LA, left atrium/atrial; LAA, left atrial appendage; RA, right atrium/atrial; RAA, right atrial appendage.

Kaplan–Meier curves and estimates of AA recurrence-free survival within 12 months after AFCA, stratified by the dichotomized atrial volumetrics, are presented in Figure 2 and Supplementary Table S2. All dichotomized volumetrics demonstrated statistically significant differences in AA recurrence-free survival within 12 months after AFCA, as determined by the log-rank test. The most pronounced difference was observed for LAA volume, where patients with LAA volume <15.3 mL had a freedom from AA recurrence rate of 0.825 (95% CI, 0.766–0.888), compared to 0.568 (95% CI, 0.439–0.735) for those with LAA volume ≥15.3 mL (log-rank *P* < 0.001).

### 3.3 Impact of various atrial volumetrics on the recurrence after AFCA

**Figure 3:**
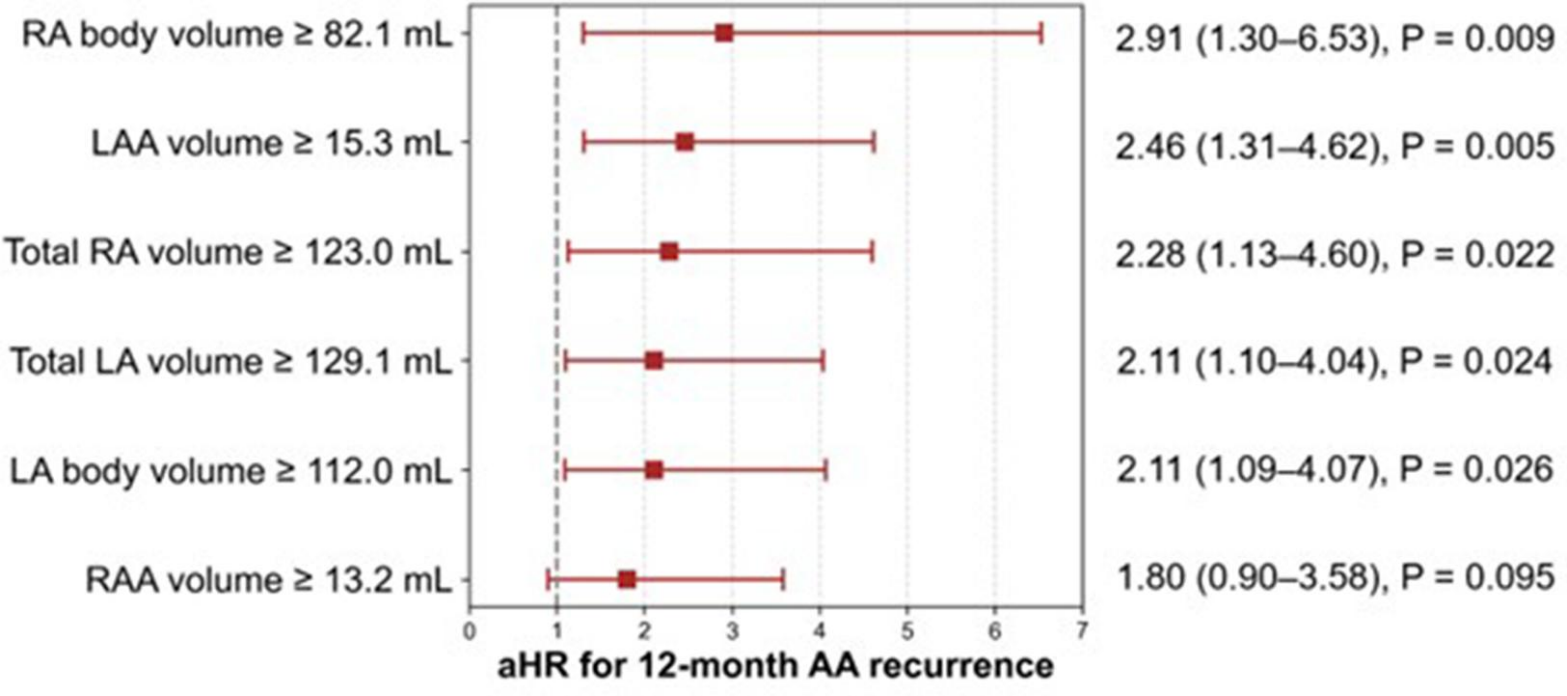
Adjusted hazard ratios (model 3) of dichotomized atrial volumetrics for AA recurrence after AFCA. AA, atrial arrhythmia; AFCA, catheter ablation for atrial fibrillation; LA, left atrium/atrial; LAA, left atrial appendage; RA, right atrium/atrial; RAA, right atrial appendage; aHR, adjusted hazard ratio.

The results of the 12-month AA recurrence risk estimation, based on dichotomized atrial volumetrics and analyzed using Cox proportional hazards models, are summarized in Figure 3 (Model 3) and Supplementary Figure S2 (Models 1 and 2). In the unadjusted model, the volumetric associated with the highest HR was LAA volume ≥15.3 mL (HR 2.74; 95% CI, 1.51–4.95), followed by RA body volume ≥82.1 mL (HR 2.48; 95% CI, 1.23–5.01) (Supplementary Figure S2).

After adjustment (Model 3), all dichotomized atrial volumetrics, except RAA volume ≥13.2 mL, were significantly associated with an increased risk of AA recurrence within 12 months after AFCA. The adjusted HRs (aHRs) with 95% CIs were as follows: total LA volume ≥129.1 mL, aHR 2.11 (1.10–4.04); LA body volume ≥112.0 mL, aHR 2.11 (1.09–4.07); and LAA volume ≥15.3 mL, aHR 2.46 (1.31–4.62). For the RA volumetrics, the adjusted aHRs were 2.28 (1.13–4.60) for total RA volume ≥123.0 mL, 2.91 (1.30–6.53) for RA body volume ≥82.1 mL, and 1.80 (0.90–3.58) for RAA volume ≥13.2 mL (Figure 3).

**Figure 4:**
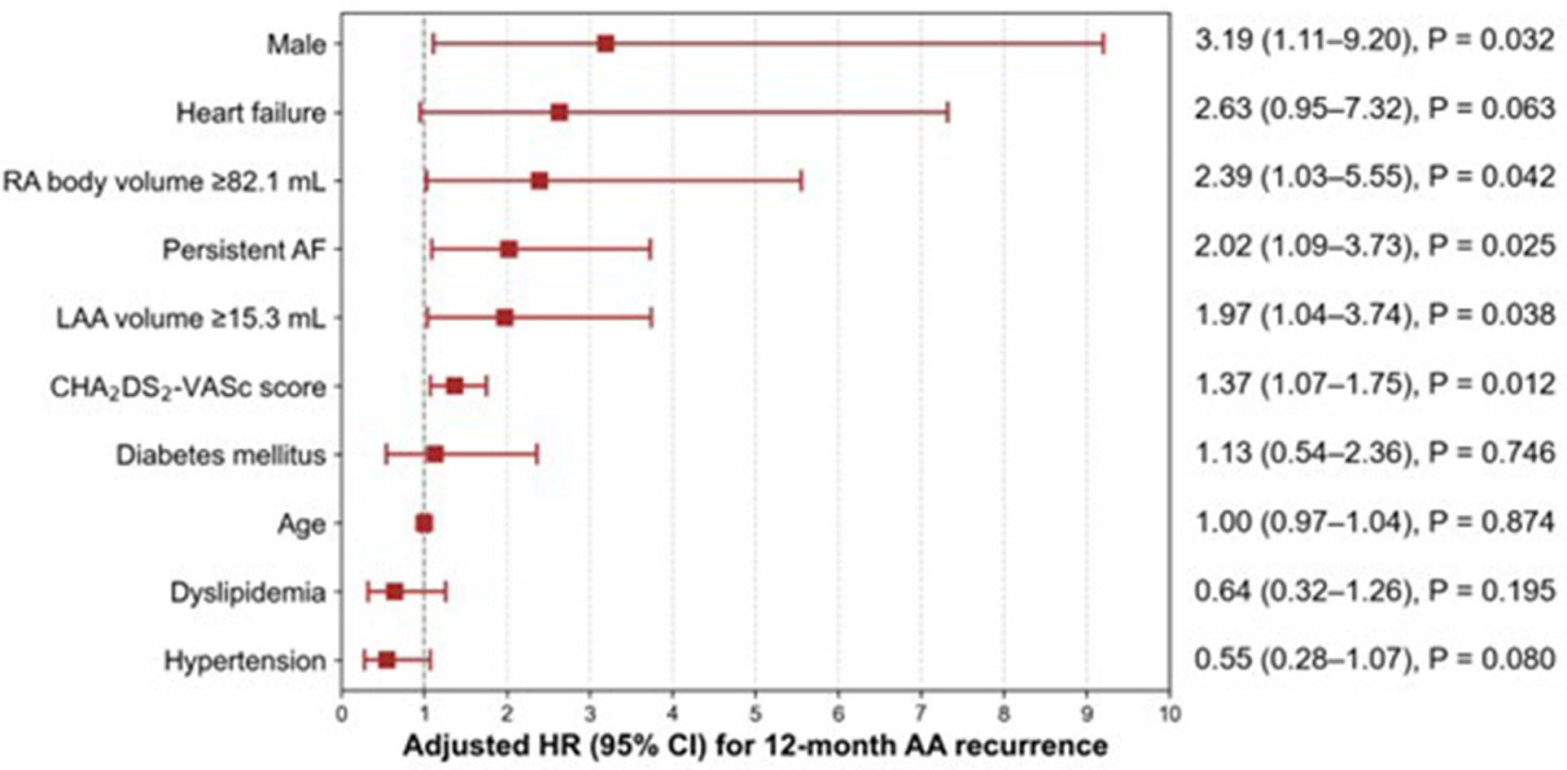
Multivariate Cox regression analysis including dichotomized LAA volume and RA body volume for AA recurrence after AFCA. LAA, left atrial appendage; RA, right atrium; AA, atrial arrhythmia; AFCA, catheter ablation for atrial fibrillation; AF, atrial fibrillation; aHR, adjusted hazard ratio; CI, confidence interval.

Finally, a multivariable Cox regression model was constructed, including LAA volume ≥15.3 mL and RA body volume ≥82.1 mL (both selected based on the highest adjusted HRs among LA and RA volumetrics), adjusted for the same covariates as in Model 3 (Figure 4). In this model, both LAA volume ≥15.3 mL (aHR 1.97; 95% CI, 1.04–3.74; *P* = 0.038) and RA body volume ≥82.1 mL (aHR 2.39; 95% CI, 1.03–5.55; *P* = 0.042) remained significant predictors of AA recurrence within 12 months. No significant multicollinearity was observed among the covariates (all VIF <5), and the model did not violate the proportional hazards assumption (all Schoenfeld *P* > 0.05).

Additional penalized Cox regression using the LASSO method was performed to evaluate model robustness. In the LASSO model using the optimal penalty parameter, both LAA volume ≥ 15.3 mL and RA body volume ≥ 82.1 mL were retained, consistent with the findings of the primary multivariable analysis (Supplementary Table S3).

## 4 Discussion

The main findings of this study were that atrial volumetrics, except for RAA volume, were independently associated with 12-month AA recurrence following AFCA. Among these, dichotomized RA body and LAA volumes were the volumetric measures most strongly associated with an increased risk of AA recurrence. To the best of our knowledge, this is the first study to propose optimal cutoff values for predicting AA recurrence using all atrial volumetrics, including total LA, LA body, LAA, total RA, RA body, and RAA volume, and to validate the prognostic utility of dichotomized volumetrics.

Consistent with previous findings, we confirmed that larger LA volume is significantly associated with recurrence after AFCA (21). This is primarily attributed to atrial structural remodeling that accompanies AF (22), which is histologically characterized by interstitial fibrosis, myocyte hypertrophy, and reduced connexin expression (23, 24). In addition to structural changes, atrial electrical remodeling also occurs and may contribute to AF recurrence (25). Furthermore, we demonstrated that a larger LAA volume was also associated with an increased risk of AA recurrence within 12 months, and it yielded the highest risk for AA recurrence among the dichotomized atrial volumetrics. This finding aligns with several previous studies that have reported a similar association (20, 26). Prior studies have also suggested that remodeling occurs in the LAA, with histological features similar to those observed in LA remodeling (27, 28).

Another notable finding was that larger total RA volume and larger RA body volume were significantly associated with AA recurrence, whereas larger RAA volume was not. As demonstrated in several previous studies, atrial remodeling in AF is not confined to the LA, but the RA also undergoes structural and electrical remodeling (11, 29–31).

The association between larger RA body volume and increased AA recurrence may be explained by several mechanisms. First, RA enlargement may reflect more advanced atrial cardiomyopathy. Atrial cardiomyopathy is a structural and electrophysiological adverse remodeling of the atria, characterized by atrial fibrosis and mechanical or conduction abnormalities that increase the risk of AF (32). Recent evidence also suggests that more advanced atrial cardiomyopathy is associated with an increased risk of AF, supporting the concept that atrial enlargement may represent increased arrhythmogenic substrate (33). Also, significant tricuspid regurgitation can aggravate atrial cardiomyopathy and lead to RA dilatation (34). Therefore, increased RA body volume can be a characteristic feature of advanced atrial cardiomyopathy and thus increases the risk of AA recurrence. Second, RA body enlargement can be a signal of a greater burden of non-pulmonary-vein triggers from the RA. Santangeli et al. reported that non-pulmonary-vein triggers from the RA become increasingly prevalent as AF progresses, particularly in the crista terminalis, coronary sinus, and right interatrial septum (35). This finding is consistent with the observation that adjunctive RA ablation in persistent AF may reduce recurrence risk (36), further supporting the clinical importance of enlarged RA in AF ablation. Third, a larger RA body may also predispose to macro-reentrant atrial tachyarrhythmias by facilitating larger reentrant circuits and slow or heterogeneous conduction (37, 38). All these mechanisms may explain why RA body volume was associated with recurrence in the present study.

Regarding the RAA, previous reports have suggested that it may serve as a potential arrhythmogenic source in AF (39, 40). However, in our study, RAA volume was not associated with AA recurrence. One possible explanation is that RAA-related arrhythmogenicity may depend more on specific anatomical characteristics than on appendage volume alone. Pan et al. reported that anatomical features, such as increased RAA height and a shorter RAA base diameter, were associated with AF recurrence after AFCA (41). Therefore, our findings do not exclude a potential role of the RAA in AF pathophysiology, but suggest that simple volumetric enlargement of the RAA may be insufficient to represent its arrhythmogenic role. Further studies integrating RA and RAA volumetric assessment, detailed anatomical characterization, and substrate mapping are warranted to clarify the role of both RA and RAA in AF recurrence.

Furthermore, we validated another multivariable Cox regression model for predicting 12-month AA recurrence after AFCA, which included both LAA volume ≥15.3 mL and RA body volume ≥82.1 mL. In this model, both larger LAA volume (*P* = 0.038) and larger RA body volume (*P* = 0.042) were independently associated with 12-month AA recurrence. These findings suggest that LAA volume ≥15.3 mL and RA body volume ≥82.1 mL may serve as a potential predictor of AA recurrence. The robustness of the observed associations was further supported by the penalized regression analysis, in which both larger LAA volume and larger RA body volume remained retained variables despite coefficient shrinkage.

The clinical relevance of this study lies in its implications for early rhythm control (ERC). ERC refers to the initiation of rhythm control strategies, including anti-arrhythmic drug therapy, electrical cardioversion, or catheter ablation, within the early phase following AF diagnosis (typically within 1 year) (42, 43). Previous studies have demonstrated that ERC is associated with a reduction in adverse cardiovascular outcomes, including ischemic stroke, cardiovascular death, and acute coronary syndrome (44, 45). From the perspective of atrial remodeling, ERC may attenuate structural and electrical changes by facilitating earlier restoration of sinus rhythm compared to usual care (43). Considering that our findings showed an association between larger atrial volumetrics, which reflect atrial remodeling, and increased risk of AA recurrence after AFCA, these results support the consideration of ERC in AF patients to improve post-ablation outcomes.

### 4.1 Limitations

This study had several limitations. First, the retrospective design limited the ability to establish a causal relationship between atrial volume and AA recurrence. Second, this study was conducted in a single center and included a relatively small sample size (*N* = 199), which may limit the generalizability of the findings. Further multicenter studies with a larger population are needed to clarify the associations. Third, although exclusion criteria were limited to objective imaging-related factors independent of clinical outcomes, the possibility of selection bias inherent to retrospective designs cannot be entirely excluded. Fourth, although the artificial intelligence-based heart chamber segmentation software was highly automated, manual delineation was required for PVs, SVC, IVC, and atrial appendages. These steps might have involved a degree of subjectivity. However, to minimize bias, the investigator was blinded to the participants' clinical information and recurrence status while delineating. In addition, the segmentation accuracy of AutoSeg-H has not yet been formally validated against expert manual segmentation in a dedicated validation study. Therefore, although all segmentation results underwent manual review and correction under the supervision of an experienced cardiologist, the precision and generalizability of the software warrant further validation. Fifth, this registry included only patients who underwent radiofrequency catheter ablation. Thus, the findings may not be generalizable to patients treated with cryoballoon ablation or pulsed-field ablation. Further studies are warranted to evaluate whether the observed associations are consistent across different ablation modalities. Sixth, procedural variations among operators may have influenced the clinical outcomes. However, as this was a single-center registry from a tertiary referral hospital, all procedures were performed according to relatively standardized institutional protocols, which may partially mitigate inter-operator variability. Also, the follow-up duration of 12 months was relatively short. Nevertheless, a previous meta-analysis including 5,710 AF patients who underwent catheter ablation reported that approximately 60% of 5-year AF recurrences occurred within the first 12 months (46). It suggests that a 12-month endpoint is reasonably efficient. Lastly, although all documented ECG recordings were used to assess recurrence, most assessments were based on 10-second 12-lead ECGs in outpatient clinic visits. The accuracy of recurrence detection could potentially be improved with daily ECG monitoring using wearable devices. While several wearable devices to acquire ECG have been developed, they were not widely used in South Korea during the follow-up duration of this study.

## 5 Conclusion

Among the atrial volumetrics, LAA and RA body volumes are the most significant independent predictors of 12-month AA recurrence after AFCA. The results demonstrate that remodeling of not only LA but also LAA and RA body sizes may influence the risk of AA recurrence after AFCA.

## Data Availability

The data analyzed in this study is subject to the following licenses/restrictions: The datasets used during the current study are available from the corresponding author on reasonable request. Requests to access these datasets should be directed to choiek17@snu.ac.kr.
