# Supplementary material for: Atrial volumetrics and recurrence after catheter ablation for atrial fibrillation
Source: Front Cardiovasc Med. 2026 Jun 22;13:1843231. doi: 10.3389/fcvm.2026.1843231 (PMC13333398; doi:10.3389/fcvm.2026.1843231)
Supplement: Supplementary file 1 [file Datasheet1.docx]

Supplementary Materials

# Supplementary Methods

## Detailed Protocols for AFCA

The detailed steps of the AFCA protocol varied depending on the study registry each patient was enrolled in, as the study population was derived from three different registries. For patients in the OPTIMUM phase 1 study, contact force-guided ablation was performed, with a target contact force of 5–20 g and an RF application duration of 20–40 s at each point. In contrast, patients in the OPTIMUM phase 2 study, the LESS study, or the TAILOR study underwent ablation index-guided ablation. Radiofrequency energy was applied to achieve an ablation index of 450-500 at the anterior and roof segments, and 350-400 at the posterior, inferior, and carina segments.(1-3)

# Supplementary Figures and Tables

## Supplementary Figures


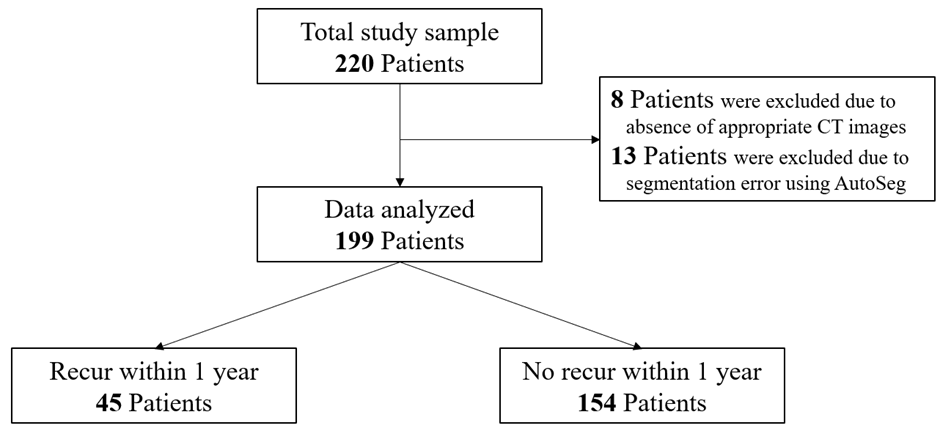


**Supplementary Figure S1.** Study flow.

Abbreviation: *CT* computed tomography.

**(A)**


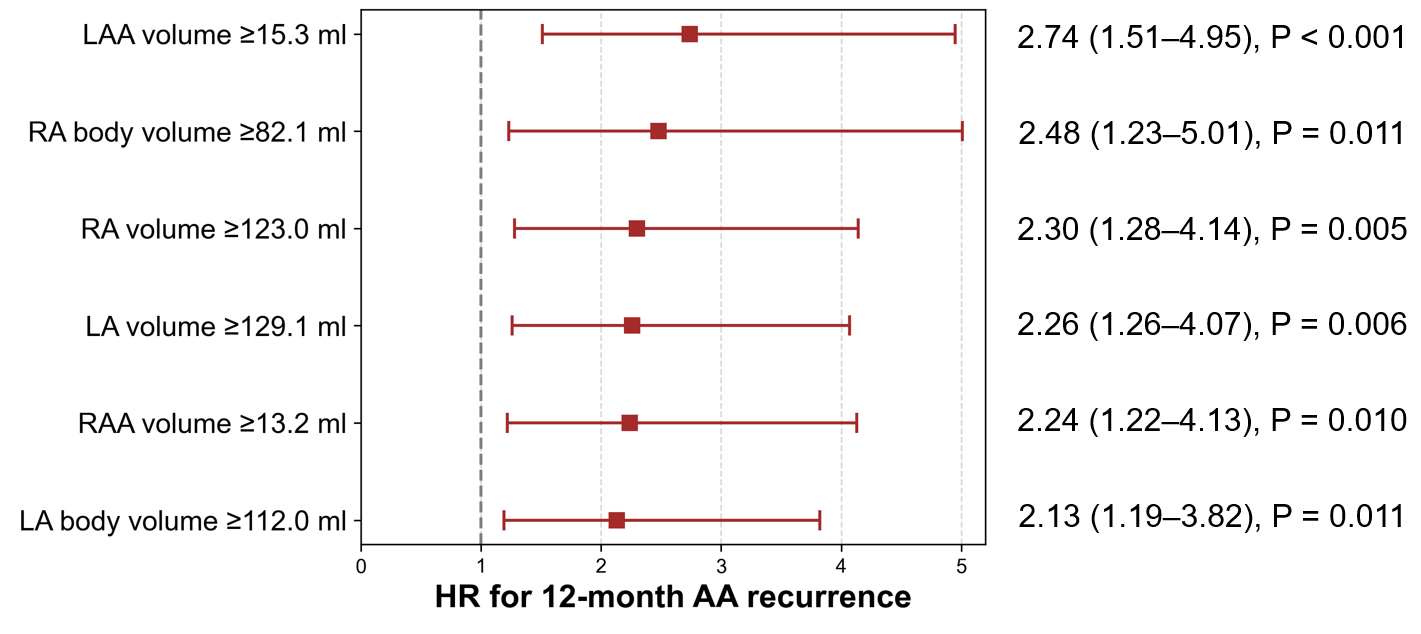


**(B)**

**
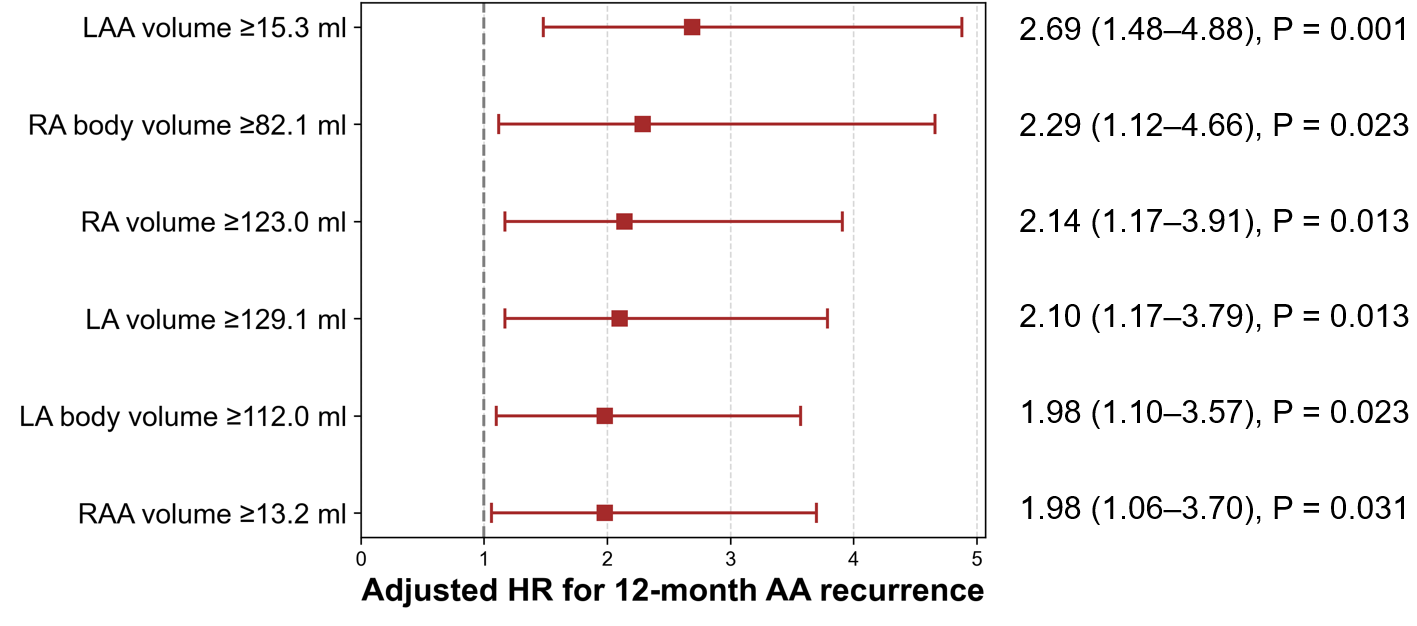
**

**Supplementary Figure S2.** Hazard ratio for dichotomized atrial volume parameters for AA recurrence within 12 months after AFCA. **(A)** Unadjusted hazard ratio (Model 1). **(B)** Adjusted for age and sex (Model 2).

Abbreviations: *AA* atrial arrhythmia, *AFCA* atrial fibrillation catheter ablation, *LA* left atrium/atrial, *LAA* left atrial appendage, *RA* right atrium/atrial, *RAA* right atrial appendage, *HR* hazard ratio, *aHR* adjusted hazard ratio, *CI* confidence interval.

## Supplementary Tables

**Supplementary Table S1.** Area under curve estimates and optimal cutoff values in ROC curves predicting AA recurrence within 12 months after AFCA using atrial volume parameters

| **Atrial volume parameters** | **Area under ROC curve (95% CI)** | **Optimal cutoffs^*^** |
| --- | --- | --- |
| **LA volume (mL)** | 0.616 (0.525–0.708) | 129.1 |
| **LA body volume (mL)** | 0.609 (0.518–0.700) | 112.0 |
| **LAA volume (mL)** | 0.598 (0.496–0.701) | 15.3 |
| **RA volume (mL)** | 0.638 (0.550–0.726) | 123.0 |
| **RA body volume (mL)** | 0.623 (0.535–0.712) | 82.1 |
| **RAA volume (mL)** | 0.609 (0.514–0.703) | 13.2 |

^*^ Optimal cutoffs are defined using Youden indices.

Abbreviations: *ROC* Receiver Operating Characteristic, *AA* atrial arrhythmia, *AFCA* catheter ablation for atrial fibrillation, *CI* confidence interval, *LA* left atrium/atrial, *LAA* left atrial appendage, *RA* right atrium/atrial, *RAA* right atrial appendage.

**Supplementary Table S2.** Kaplan-Meier estimation for freedom from AA recurrence within 12 months after AFCA

| **Atrial volume parameters** | **Freedom from AA recurrence**  **(95% CI)** | **Log-rank P-value** |
| --- | --- | --- |
| **LA volume**  **<129.1 mL**  **≥129.1 mL** | 0.824 (0.762–0.890)  0.629 (0.514–0.768) | 0.005 |
| **LA body volume**  **<112.0 mL**  **≥112.0 mL** | 0.826 (0.762–0.895)  0.653 (0.548–0.779) | 0.010 |
| **LAA volume**  **<15.3 mL**  **≥15.3 mL** | 0.825 (0.766–0.888)  0.568 (0.439–0.735) | <0.001 |
| **RA volume**  **<123.0 mL**  **≥123.0 mL** | 0.828 (0.766–0.894)  0.628 (0.515–0.764) | 0.004 |
| **RA body volume**  **<82.1 mL**  **≥82.1 mL** | 0.868 (0.795–0.948)  0.700 (0.621–0.788) | 0.009 |
| **RAA volume**  **<13.2 mL**  **≥13.2 mL** | 0.808 (0.748–0.874)  0.609 (0.476–0.778) | 0.008 |

Abbreviations: *AA* atrial arrhythmia, *AFCA* catheter ablation for atrial fibrillation, *CI* confidence interval, *LA* left atrium/atrial, *LAA* left atrial appendage, *RA* right atrium/atrial, *RAA* right atrial appendage.

**Supplementary Table S3.** LASSO penalized Cox regression analysis for predictors of AA recurrence after AFCA.

| **Variable** | **LASSO coefficient** | **Retained** |
| --- | --- | --- |
| LAA volume ≥ 15.3 mL | 0.624 | Yes |
| RA body volume ≥ 82.1 mL | 0.656 | Yes |
| Male | 0.792 | Yes |
| Age | 0 | No |
| Persistent AF | 0.601 | Yes |
| Heart failure | 0.775 | Yes |
| CHA_2_DS_2_-VASc score | 0.216 | Yes |
| Diabetes mellitus | 0.020 | Yes |
| Dyslipidemia | -0.249 | Yes |
| Hypertension | -0.409 | Yes |

Abbreviations: *AA* atrial arrhythmia, *AFCA* atrial fibrillation catheter ablation, *LAA* left atrial appendage, *RA* right atrium, *AF* atrial fibrillation.

# References

1. Lee S-R, Choi E-K, Lee E-J, Choe W-S, Cha M-J, Oh S. Efficacy of the optimal ablation index–targeted strategy for pulmonary vein isolation in patients with atrial fibrillation: the OPTIMUM study results. Journal of Interventional Cardiac Electrophysiology. 2019;55:171-81.

2. Lee SR, Park HS, Choi EK, Lee E, Oh S. Acute and long‐term efficacy of ablation index‐guided higher power shorter duration ablation in patients with atrial fibrillation: A prospective registry. Journal of Arrhythmia. 2021;37(5):1250-9.

3. Lee SR, Park HS, Kwon S, Choi EK, Oh S. Tailored ablation index based on left atrial wall thickness assessed by computed tomography for pulmonary vein isolation in patients with atrial fibrillation. Journal of Cardiovascular Electrophysiology. 2023;34(9):1811-9.
